# *“As we have gathered with a common problem, so we seek a solution”*: exploring the dynamics of a community dialogue process to encourage community participation in family planning/contraceptive programmes

**DOI:** 10.1186/s12913-019-4490-6

**Published:** 2019-10-17

**Authors:** Tamaryn L. Crankshaw, Yolandie Kriel, Cecilia Milford, Joanna Paula Cordero, Nzwakie Mosery, Petrus S. Steyn, Jennifer Smit

**Affiliations:** 1 Health Economics and HIV and AIDS Research Division (HEARD), University of KwaZulu-Natal, Durban, South Africa; 2 MatCH Research Unit (MRU), Department of Obstetrics and Gynaecology, Faculty of Health Sciences, University of Witwatersrand, Durban, South Africa; 3 UNDP/UNFPA/UNICEF/WHO/World Bank Special Programme of Research, Development and Research Training Human Reproduction, Avenue Appia 20, 1202, Geneva, Switzerland

**Keywords:** Community participation, Health provider, Family planning, Contraception, Unmet need

## Abstract

**Background:**

Community dialogues have been widely used as a method for community engagement and participation to cover a broad range of areas. However, there has been limited documentation and evaluation of the process, particularly as a method towards achieving family planning and contraception (FP/C) programme goals. As part of the development of an intervention package aimed at increasing community and health care provider (HCP) participation in the provision of FP/C, feasibility testing of the intervention approach (a community dialogue between communities and health facilities) was carried out. Our findings offer a systematic description and evaluation of the community dialogue process, with key recommendations towards future implementation.

**Methods:**

The dialogue was evaluated in three ways: 1) through participant observation during the community dialogue, 2) via a standardised feasibility testing tick-list for all observers of the dialogue, and 3) through three focus group discussions (FGDs) consisting of different groups of stakeholders who participated in the community dialogue. In total, 28 community members, HCPs, and key stakeholders attended the community dialogue (22 females, 6 males). Twenty-seven of the community dialogue participants participated in one of 3 FGDs held after the dialogue. Six evaluators assessed feasibility of the dialogue process.

**Results:**

There was good attendance, representation and participation amongst community and provider sectors based on the participant observations using the standardized feasibility check-list. The community dialogue process received positive feedback in the FGDs and was demonstrated to be feasible and acceptable. Key factors contributing to the success of the community dialogue included a skilled facilitator, good representation of participants, establishing ground rules, good timekeeping, and using a Theory of Change to facilitate goal identification and dialogue. Issues to consider are the underlying power differentials related to age, profession and gender which caused initial feelings of anxiety amongst some participants.

**Conclusions:**

Our formative findings offer a systematic description and evaluation of a community dialogue process with key recommendations that may be considered when constituting similar community dialogues in the future.

**Electronic supplementary material:**

The online version of this article (10.1186/s12913-019-4490-6) contains supplementary material, which is available to authorized users.

## Background

The concept of communities being empowered to take ownership over their own health through their participation in the development of health strategies, policies and interventions has long been endorsed by international bodies and public health agencies [1–3]. The notion of ‘community participation’ underscores a human rights approach to health by working towards reducing health disparities and by improving quality of services through increased collaboration between health care providers (HCP) and the community members they serve [4–6]. Of key importance is the role that community participation has in promoting social cohesion - which is the level of interconnectedness and unity amongst groups in society, and which has long been linked to community level well-being and health [7, 8].

While there is broad consensus over the value of participatory approaches, the actual implementation is complex and challenging to execute. That is, the concept of ‘community’ encompasses a wide range of actors within which not everyone will be viewed equally or empowered to participate [9]. This is especially salient when there are gendered, socioeconomic and class differentials which typically exist between HCPs and community members. Indeed, previous health efforts adopting a community participation approach found that unequal power relationships between community members and HCPs fundamentally impeded successful participation [10].

While community participation is viewed as a key component of family planning and contraceptive (FP/C) programming [11, 12], lack of consensus around how this should be approached and differing definitions as to what constitutes ‘community participation’ have hamstrung efforts to meaningfully integrate this approach in national family planning programmes [6]. An additional challenge to participation in family planning programmes are the cultural sensitivities around family planning and the overt focus of family planning on prevention [13, 14]. More broadly, participation has been successfully applied to the domain of maternal and child health but these successes are less evident in large scale family planning programmes [15]. A recent review reporting the mixed impact of differing approaches to promoting participation between community and healthcare providers in FP/C programmes identified one promising approach [16]. Specifically, interventions which created a forum for dialogue between the differing stakeholders have seen improvements in quality and accountability of programmes [16].

This paper is based on formative research for a proposed multi-country (Kenya, South Africa and Zambia) study (the UPTAKE project), with the aim of increasing met need for FP/C, through the development and testing of an intervention involving community and HCP participation within a human rights framework. The intervention design was guided by a theory-driven process to conceptualise the underlying mechanisms of change that the intervention sought to achieve (Theory of Change) [17–19]. The advantages of using Theory of Change (ToC) in the evaluation and unpacking of complex interventions such as those dealing with health have been explicated [17, 20, 21]. ToC is a theory driven approach to evaluation which enables the identification of possible causal pathways, as well as the requirements and assumptions that are needed to be fulfilled in order to achieve programme goals [17]. Additionally, development and use of ToC frameworks is participatory and iterative [17]. The ToC underlying the UPTAKE intervention design hypothesised that if an approach could be created to promote effective participation between the community and HCPs, this would increase trust and better understanding of respective perspectives and barriers to FP/C services [22, 23], as well as support co-identification of appropriate actions and solutions [22]; this would then lead to improved service provision which would increase service utilisation, service satisfaction, and method continuation, as well as empower clients. Accomplishment of these intermediate outcomes, it was hypothesised, would work to reduce unmet need for FP/C and risk of method discontinuation.

Quality of Care (QoC) – one of the nine human rights standards [24] - was identified as a possible domain that could pose barrier to access to services; this was based on a literature review and working group consultation consisting of experts in family planning, human rights and participation, as well as the UPTAKE project principal investigators. QoC in the context of family planning may include the availability of a range of methods, evidence-based information on risks and benefits, and acceptability. The perception of the quality of contraceptive service and information provision influences use and client satisfaction. Thus QoC improvement can increase the effectiveness of sexual and reproductive health services [25]. At the beginning of the formative research, the concept of QoC was explored in focus group discussions (FGDs) and in-depth interviews with the community, health providers and other stakeholders. Based on the feedback, QoC was chosen as a sample domain during the feasibility testing of the intervention approach to encourage community participation in FP/C services. The approach took the form of a community dialogue between communities and health facilities - reported here. The structure of the community dialogue was designed and tested for acceptability and feasibility amongst the participating community. This paper presents the findings from the community dialogue piloted in Durban, South Africa. We aim to describe and provide an analysis of the community dialogue process, highlighting successful structural elements as well as presenting some underlying considerations when constituting community dialogues of this nature.

## Methods

This formative research conducted in three countries (Kenya, South Africa and Zambia) aimed to develop an intervention package for increasing community and HCP participation in the provision of FP/C. Inclusion criteria for countries were i) Either low contraceptive use, or skewed method mix, ii) A standard minimum package of methods is currently registered and available in the public health care system (barrier contraception, one short-acting method, one long acting method, emergency contraception and a permanent method in the catchment-area) and iii) A Contraceptive and Family Planning policy is in place supporting human rights principles. South Africa has high rates of unplanned pregnancy and high unmet need for contraception (15% amongst married women and 24% amongst unmarried sexually active women) [26, 27]. The study developed a ToC underpinning the intervention rationale which identified the hypothesized pathways to address unmet need for FP/C services within a human rights framework; QoC was included as one of the nine aspects of optimal service provision. The ToC was driven by a participatory approach in the form of a community dialogue. Ahead of the community dialogue, qualitative research was conducted exploring the knowledge, attitudes, and practices of utilization of FP/C services, barriers and facilitators in access to FP/C services, understandings of the concept of QoC, and activities and practices around community participation. The research findings (manuscript currently under review) informed the design of the community dialogue. The feasibility of bringing together stakeholders for the purpose of a community dialogue was then evaluated. For this, facilitator guides were developed outlining a step-by-step process for the delivery of the community dialogue and evaluation thereof. Ahead of the actual dialogue, the research team engaged in a series of preparatory meetings and conducted a dry run through the full community dialogue process with the facilitator. The dialogue was conducted in two languages: English and *isiZulu*.

The operational and cultural feasibility of the community dialogue was evaluated in three ways: 1) through participant observation during the community dialogue, 2) via a standardised feasibility testing tick-list for all observers of the dialogue, and 3) through three FGDs consisting of different groups of stakeholders who participated in the community dialogue (groups were comprised of HCPs only, community members only, and a mixed group of HCP and community members). Community members were broadly defined as possible beneficiaries, users and potential users of family planning services. The country team then consulted various stakeholders at the district level to ensure that the most inclusive definition of “community” for the particular context was achieved.

*Participant observers:* There were six participant observers, all of whom were research staff and who had qualitative methods research training. The observers assisted with time keeping, noted responses and behaviour being displayed by participants, drew the facilitator’s attention to participants who were less confident to speak up in the group and who needed to be drawn into the discussion, and supported the facilitator in the reframing of questions when conversation stalled. The observers addressed all feedback directly to the facilitator in the form of notes so as to not disturb the dynamic of the dialogue. All participant notes were transcribed and collated.

### Sample sizes

#### Community dialogue

Out of 62 participants who were invited, 32 confirmed that they would be attending. A total of 28 participants arrived on the day (87.5%). One person had not been invited or expected but, since she had accompanied an invited participant, was also included in the dialogue. Participants of the community dialogue included health care providers (*n* = 3; 2 females and 1 male), NGO and government health stakeholders (*n* = 3; 2 females and 1 male), community related stakeholders (*n* = 5; 5 females), community members (*n* = 17; 13 females and 4 males). Community members included 1 female sex worker and 2 female adolescents, both aged 17. In total, the community dialogue consisted of 22 female and 6 male participants. The duration of the dialogue was 3 and a half hours with a 10 min tea break after the first hour.

#### Focus group discussions

There were 27 participants in total in the FGDs; participants were selected based on convenience into the groups. Eleven people participated in the mixed FGD (8 females, 3 males), 11 people participated in the community group (8 females, 3 males) and 5 in the HCP group (4 females, 1 male). The FGDs lasted between 1 and 1.5 h.

The purpose of these FGDs was to gain feedback on the feasibility and acceptability of the concept of using a ToC methodology in the context of a community dialogue. In addition, we examined the data to explore whether the community dialogue offered the anticipated enabling/empowering platform for participation between community members and HCPs and if so, what factors facilitated the success.

### Recruitment processes

#### Community dialogue

Participants for the community dialogue were recruited from the research team’s existing networks in the eThekwini area. Health care provider participants were recruited from health facilities. Identification of health facilities began in 2015 for the purpose of the ethics submission. At the project inception, a team brainstorming session was conducted where all the health care facilities operating under the KwaZulu-Natal Provincial Department of Health were identified. The facilities falling into the planned second phase, intervention areas were excluded from the community dialogue recruitment process. Two facilities, which fell outside of the intervention sites, were identified for the purpose of recruiting HCPs into the community dialogue. These two health facilities were approached by project team members and informed about the planned study to gain buy-in for the event. One staff member later returned to each facility and issued invitation letters and information sheets to each of the operational managers with a view to recruiting health care providers. Other relevant stakeholders were identified through brainstorming with the project team, drawing on existing research networks. Electronic invitations were sent out to most stakeholders. In addition, a staff member visited various civil society facilities in person to invite further stakeholders. The Community Advisory Board (CAB) established at the study investigators’ research office were contacted to assist with the recruitment of community members. CAB members were included as part of the stakeholder group.

#### Focus group discussions

Participants who were involved in the community dialogue were provided lunch and then invited to participate in the FGDs directly afterwards (please see Additional file 1 for the interview guide). All FGDs were audio recorded, transcribed and translated (in the case of the community and mixed FGD).

### Ethics

Information sheets accompanied all invitations. Minors were provided with information sheets for their parents to read and a consent form requiring parental signatures to allow them to participate in the FGDs. Assent was also obtained from all minors. Written informed consent was obtained from all FGD participants. Ethical approval was obtained from the University of the Witwatersrand’s Human Research Ethics Committee (HREC) [approval number M1504101] and the WHO’s Research Ethics Review Committee (WHO ERC).

## Results

### Community dialogue process

There were three main components to the community dialogue: 1) Orientation, 2) Establishing ground rules, 3) Elucidating the ToC approach. The orientation section included welcoming participants and clarifying expectations for the dialogue. Participants were asked to pair off, introduce themselves to each other and then introduce the other to the group. While this took time to complete, it proved to be a successful ice-breaker where participants visibly settled and focussed on the proceedings. Participants’ expectations for the day were slightly misaligned with the goal of the dialogue in that they expected to learn more about FP/C (passive role) rather being seen as key contributors to the discussion (active role). This provided an opportunity for the facilitator to clarify expectations and the purpose of the dialogue.

Establishing ground rules involved introducing the concept of “ground rules” and asking participants to identify their own ground rules, while still ensuring that these covered the intervention’s predefined ground rules. Participants had very clear ideas about what should constitute these rules and there was high agreement between participants in establishing the ground rules. These included respect for people’s opinions, to project voices, set phones on silent, try to express points clearly, raise hands if they wanted to talk, no side meetings, to participate actively, stay on topic, and acknowledge all were equal in the group, including younger participants.

The third component of the dialogue consisted of presenting the ToC which was used to structure the dialogue by providing a framework to identify the problem as well as the desired outcome. The dialogue was then presented as a tool to identify steps and actions that need to occur to achieve the desired goal (in this case, QoC was chosen as the key intermediate outcome). To introduce this section, the facilitator posed a series of questions, for example “Why is unmet FP need a problem in your community?”. Participant observers noted that adolescent and male participants did not contribute to these initial discussions, and after communicating this to the facilitator, these groups were more actively drawn into the conversation. The facilitator then introduced the concept of QoC. Participant observers noted that this session needed tight time management and sophisticated facilitation skills in order to separate key QoC issues from the more “off-the-topic” information offered by participants.

The dialogue was led by an experienced female facilitator fluent in both English and *isiZulu* (local language). The facilitator actively engaged HCPs, community participants and key stakeholders in the discussion so that all perspectives were presented. Participants found it challenging to answer the question, “What can you do to improve quality of care?” and were more able to identify what the problems with FP/C were. HCPs were initially withdrawn from engaging in discussions related to how QoC could be improved and community members displayed interest in hearing HCP perspectives. Participants started to visibly tire after 2 h and 20 min and the dialogue was drawn to a close. The following section presents the findings from the focus group discussions evaluating the community dialogue process.

### Focus group discussion findings

#### Inclusion and representation in the community dialogue

Participants broadly perceived that the community dialogue was representative of the ‘community’:

Female (29 years): *I think that everybody was there because those that we needed the most are from [the Department of] Health. They are the ones who are going to play a major role in [implementing] the solutions that we, as the community, offered. Because we can speak and say we need this, and that has to be in hospitals and clinics. And people representing Health were there [to hear us]*.

Facilitator: *Do you think that all groups that are in the community in our district are represented in our community dialogue that we had?*

Female (40 years): *Yes, because there was everyone.... There were community people and there were clinic assistants [healthcare providers] and children*. (Mixed FGD)

A number of participants also identified the need to include younger adolescents of school going age and raised the linked issue of holding the dialogue during school holidays or over the weekend to accommodate inclusion of younger adolescents.

*… as much as there was youth and health personnel and so on, I felt that [age] 14 and 15 were not there and I felt that most of the time while we were there, we focused mainly on young people who are at schools. So I felt that the other side, especially of school children, was not represented. Because most of us who were down there have finished school.* (Mixed FGD, Male, 22 years)

Other participants observed value in including more men:

*The dialogue should include older men due to ‘sugar daddies’, also educators, the church can serve as a barrier due to religious beliefs, taxi drivers are out there and in contact with the young girls*. (HCP FGD, Male 1, age unknown)

As suggested above, custodians of social and religious customs and norms were also noted as missing, although there were mixed views in including them for fear of stifling conversation.

*… we need to be cautious about who we include, for example, if we include a senior pastor in the same room as a 15 year old, this won’t allow the 15 year old to be free with his/her views. We need to look at the setting and target group and how to get them together so we don’t breach beliefs. We need to ensure an appropriate platform in order for it to lead to positive results. Pay attention to the target.* (HCP FGD, Female 1, age unknown)

#### Power differentials within the group

As suggested above, the FGDs revealed the presence of pervasive underlying power differentials related to age, profession and gender – which caused initial feelings of anxiety amongst some community dialogue participants. As one young participant reflects:

*… I was shocked because the topic we were talking about … there were older people! You see [the discussion was going to include] maybe things that you will not be comfortable to talk about in front of your parent. The people that were there were in my parent’s age group. But as time went on, I could see that they were open.* (Community FGD, Female, 20 years)

Another participant in the same focus group added:

*… we were not of the same age group and there was the community and, like, they are less [have lower status] than the health.* (Community FGD, Female, 41 years)

While awareness of the power differentials was a source of anxiety for some participants, for the above and other participants, the community dialogue was seen as an opportunity to be heard.

*I was looking forward to coming to the dialogue because most of the time us women, we are silenced. So I was happy that my voice would be heard*. (Community FGD, Female, 41 years)

A younger participant shared that he had been anxious at the outset of the dialogue because there were older people in the room, however, these feelings abated as the dialogue progressed:

*[W] hen I arrived I was afraid because there were older people [ … ]. It was my first time to come and talk with so many people, [ … ], where they debate. As time went on I felt comfortable; I ended up answering because I could see that everyone was open.* (Community FGD, Male, 20 years)

The underlying power differentials between health care providers and community members were highlighted by a number of participants and these underscore the complexities in bringing together these groups to dialogue on a shared health concern.

*As I arrived I saw people, in fact people from health - they usually feel uncomfortable if they are going to be told what they are doing … so there is that thing that maybe we won’t be able to say directly what we don’t like … but as time goes on I then saw ok they are also these people who are ready to [hear].* (Community FGD, Female, 41 years)

This concern was echoed by a HCP and demonstrates that careful attention needs to be paid to the facilitation of the dialogue so that a platform is established that is conducive to constructive discussion:

*I felt so comfortable; the community did not offend us as HCPs. They put forward their points as facts. We can take these points back to our clinic and try implement them. We are working in the clinic and we don’t fully understand what some of the problems are. We usually just overhear the teenagers talking loudly and complaining but we don’t actually know what the problem is*. (HCP FGD, Female 5, age unknown)

A community member in a separate focus group confirmed that:

*There were no attacks between the community and health care providers. We had a successful discussion and negotiated differing points of view without judgement. There was no “community team” and no “healthcare provider team” who took up an aggressive position against one another.* (Mixed FGD, Male, 25 years)

The fact that HCPs are also members of their community was raised – this can be either a strategic opportunity or a barrier to free conversation if HCPs are invited from the same community. The following individual who joined the dialogue as a community member had a former professional relationship with the clinic:

*I was in a tight spot especially given the fact that there were people from health where I was working. It was going to seem as if now I’m bashing them for some reason, honestly there are things that are there at the clinics that I couldn’t have revealed because I was in a position where these people, I was working with them. It will appear as if now that I have stopped working with them, I’m now putting them down or something, so well if I could go to another dialogue in a different place I could say that and that.* (Community FGD, PID unknown)

#### Enabling and transformative platform

The community dialogue proved to be an enabling platform on several levels. Participants reflected that it had been an informative space and one in which personal learning had occurred:

*You find that sometimes you go there to take family planning but down the line [over time] you stop. You don’t understand what made you stop. Or you understand that maybe you have been influenced by certain people. They tell you about the injection, that it does 1, 2 and 3, but [then you] never do a follow up and you never go to the clinic to find out what causes these side effects. … the dialogue, like this, it was an eye opener in that … things like those you sometimes take them anyhow if you are in trouble, it is where you realise what I should have done*. (Mixed FGD, Female, 48 years)

Other participants highlighted the multifaceted value of the dialogue:

*The community dialogue was a good platform on multiple levels. I am a health care provider and [government] stakeholder and a mother of three daughters; I learned a lot [in my official capacity] and it triggered a lot for me, as an individual. It raised many things for me and provided multifaceted exposure for me in all my roles*. (HCP FGD, Female 1, age unknown)

The dialogue process was particularly transformative for HCPs and held the potential for discussion to be translated into action:

*… the discussion made me realise that there is no ‘one size fits all’ approach. I remember one participant saying that it would be good to have one service on one day so that you focus on that service. This way of doing things used to be the case in the health sector and had been done away with. But people are asking for it. Another participant said that HCPs need to have enough time to speak with us and explain things. Later on a youth said they didn’t want to have to wait in the clinic for long periods of time. One size does not fit all – each community is different. The more a service provider engages with the community, the more likely we can meet their expectations and needs.* (HCP FGD, Female 1, age unknown)

Further, participant comments highlighted that the discussion had fomented a sense of cohesion between community members and HCPs.

*...this process has taught me the power of working together, as community members and health care providers.* (HCP FGD, Female 2, age unknown)

Interestingly, the dialogue also provided leverage for one HCP to institute change.

*I can give feedback [to my colleagues] and further emphasize: ‘I heard it from the horse’s mouth’.* (HCP FGD, Male 1, age unknown)

Another participant living in a rural area recounted how the dialogue was an opportunity to be exposed to other key stakeholders:

*… what I was excited about is to know how people think because we live in rural areas. We think that people are not concerned about these things yet they are.* (Mixed FGD, Female, 40 years)

At the close of the community dialogue itself, a few of the participants expressed feeling empowered through being included in the process. One young, unemployed male participant shared to the group:

*This makes us feel honoured even though we are unemployed. We can now share this information with others*. (Male participant)

A key stakeholder added:

*… as we have gathered with a common problem, so we seek a solution* (Female participant)

#### Factors which contributed to the enabling environment

We identified several key elements which contributed to balancing underlying power differentials and creating an enabling and participatory dialogue environment.

##### Introductions and establishing ground rules

Participant feedback highlighted that the introduction process was particularly successful in making participants feel at ease.

*I felt comfortable because when we started they said we should introduce each other. After that, [ … ] when we were talking I was able to answer and saw that, well these people are open - what am I going to be afraid of?* (Community FGD, Female, 18 years)

The fact that the ground rules had been elicited from participants was a key aspect to the successful process, as the following participants reported:

*The ground rules were mutually set, they were not pushed down our throats. This allowed for ownership and set the tone of the dialogue.* (HCP FGD, Male 1, age unknown)

*The rules assisted in that we are not of the same age group, you know, so it helps that we follow what has been applied, as the rule, so that we can follow on the discussion, so that our discussion can be a success.* (Community FGD, Male, 20 years)

It was clear that the establishment of mutually elicited and agreed upon ground rules was instrumental in creating an environment where health care providers were receptive to feedback from the community, and particularly from adolescents. As one health stakeholder recounts:

*[I] understood the ground rules clearly – it really assisted with a good flow of the whole dialogue. It is not easy to have people come from different professional categories or walks of life and of different ages, especially if dealing with a difficult topic like family planning. The ground rules took the discussion in a way that was smooth and productive, there were no emotional outbursts*. (HCP FGD, Female 1, age unknown)

The ground rules also established a strong basis for respectful behaviour between participants. As one health stakeholder participant reported:

*I felt comfortable because of the ground rules. The ground rules included to respect each other, as an older person, I expect respect and the ground rules allowed for this. For this reason, I didn’t feel offended by comments from younger participants, everyone felt free to participate.* (HCP FGD, Female 4, age unknown)

These sentiments of mutual respect between participants was echoed by a participant in another focus group:

*… there were no discriminatory words used to degrade anyone.* (Mixed FGD, Female, 40 years)

##### Skilled moderator

The key role of a skilled facilitator underpinned the success of the dialogue. Community members provided positive feedback over the facilitator’s approach, describing it as warm and, as with the HCP feedback, found the facilitator’s method of communication and use of concepts easily understandable.

*When I came I was afraid because there are older people but that lady there who was facilitating, she was able to make it to be open, because the way she was talking, she was open and you could see that in this room there is a respect between the people although we were of a different age group.* (Community FGD, Female, 32 years)

*...what can I say I was not afraid from the beginning because [the Facilitator] even if we were frightened, [ … ] the way she was speaking and the way she was conducting us. From, us community members to health providers. It was... what I can say -it was warm, it was just all right.* (Mixed FGD, Female, 29 years)

The facilitator employed a variety of strategies with positive results. Throughout the dialogue, the facilitator interchanged between the two local languages, and took the time to explain concepts using familiar terminology.

*Ayi words were not hard especially because you were explaining in English and in Zulu, it was easy to understand what you were saying*. (Mixed FGD, Female, 20 years)

The facilitator also offered participants the opportunity to write down questions as an alternative to speaking up:

*… it assisted us who are shy.* (Unknown PID).

The facilitator was supported by timekeepers to keep the various components of the dialogue within the predetermined time allocated.

*I noticed that [the time] was right … we were talking and not paying attention to time because sometimes we will all want to come up with the answer*. (Mixed FGD, Female, 39 years)

## Discussion

Community dialogues have been used as a method for community engagement to cover a broad range of areas over the past two decades [28, 29]. However, there has been limited documentation and evaluation of the process, particularly as a method towards achieving FP/C programme goals. The findings from our community dialogue process offer some concrete in country insights and points of action and solutions for constituting similar community dialogues in the future.

Community dialogues provide a platform for local aspirations, concerns and values to be taken into account, as well as provide a space for individuals to identify context specific problems and to be involved in developing appropriate solutions [28–30]. In principle, by working together towards a precise and acceptable target for action, community governance of their own health can be strengthened [29, 30]. The construct of social cohesion emerges from the participatory process and “links community participation with notions of trust, shared emotional commitment and reciprocity” (30:p533) among community members [8, 30, 31]. These aspects can act to empower communities by increasing social capital and feelings of collective efficacy within the community [32]. Unmet need for contraception and risk of discontinuation involves much more than just the woman concerned and, as such, collective support of FP/C utilisation is key to preventing unplanned pregnancies. Our findings highlighted the initial anxiety and surprise expressed by young community members on being encouraged to have voice in a forum which included older members of the community and adult health care providers who traditionally occupy positions of power and authority, also within the community. Indeed, all categories of participants expressed some form of anxiety and reservation, based on past experiences and normative expectations around the prospect of participating amongst a diverse group of stakeholders. Because of the power dynamics that are likely to be present when constituting a community dialogue of this nature, it is recommended that time spent orienting participants, setting objectives and expectations, conducting ice-breaker activities, and agreeing on ground rules should be a key component of the overall dialogue process. Allowing the group to set the ground rules and not displaying them in advance was viewed as a key success factor since it created ownership of the process and set the tone of the dialogue from the outset.

Our feasibility testing of a community dialogue between community members and HCPs demonstrated that the dialogue method is a promising participatory approach in engaging community members to discuss issues related to FP/C, to promote mutual understanding and create awareness of the realities, perspective and conditions of the other. Not only did our dialogue encourage those less empowered to speak up and contribute to understandings of quality of care but the very act of being included and being invited to contribute was a transformative experience for many participants. Importantly, participation helped transcend socioeconomic, gender, age and class differentials – all crucial elements of empowerment theory [30]. While the relationship between participation and empowerment is complex, participation is widely viewed as a precursor to empowerment [33–35]. Examples of this were reflected in our findings, for example, the unemployed male who, by the very act of being invited to the dialogue and his opinion sought, expressed feeling less marginalised, and the female participant appreciating the value of a platform being created where her voice could be heard. However, research on the moderating influence of gender on participation [30, 36] underscores the importance of a skilled facilitator in encouraging participation from all participants. The role of the facilitator was identified as crucial to the success of the dialogue, as was the assistance of participant observers who acted as a support to the facilitator. Future dialogues will need to ensure that the facilitator is highly skilled and well-versed in the intervention outcomes, as well as provide the facilitator with support in the form of a note taker to keep the dialogue on track and within the times allocated for the different activities. The note taker can also act as an observer and assist the facilitator by pointing out when certain groups (e.g. men, youth) fail to engage for a period of time so as to consistently encourage participation.

The findings highlighted that not only are communities diverse in composition but that community members will have overlapping identities and roles, such as being a parent, health care provider and a health sector stakeholder, for example. While a participant may choose (or be asked) to represent a particular identity at a given community dialogue, our findings indicate that the participatory process also has the potential to catalyse personal reflections and empathy, suggesting that the dialogue method may have additional benefits beyond the intervention goal. Ensuring adequate representation of community members will be key to future dialogue sessions and will require significant planning. For example, community members were easier to recruit than other stakeholders and recruiters experienced difficulties in securing enough men (compared to women) to participate, perhaps reflecting the economic dynamics and, thus, availability of these different groups [30]. The adolescent population represented the most challenging group to recruit due to the community dialogue being held during school hours. Consideration of appropriate times or days (and spaces) to hold a community dialogue will be a key factor in successful recruitment of participants to ensure inclusive representation.

It was noteworthy that the one sex worker who was present at the dialogue did not identify herself as such. Instead, she indicated she was a community member. The implications of this were that discussion of barriers to QoC, as experienced by sex workers, were not raised. Future planning for dialogues of this nature may consider ways to engage marginalised populations beyond merely ensuring presence at the dialogue. It would also be worthwhile to consider ways to hear the voices of adolescents. The participant observations noted that adolescents were attentive to the discussions but that they rarely offered their opinion unless directly asked by the facilitator. This also applied to men. Men rarely offered their opinion spontaneously but when asked directly, they had a view to share and displayed comfort in sharing this view – they just needed invitation to express it. One solution could be to give participants the option to write down their suggestions or contributions on a piece of card provided at the start of the dialogue – an approach which was successfully tested in the above community dialogue. The underlying rationale was to afford the more reserved participants an opportunity to contribute if they were not comfortable expressing their opinion to the group directly. The facilitator then read out the suggestions and prompted discussion around the issue area. This method only includes literate community members and could potentially be stigmatising for illiterate community members. However, if presented to the participants as an optional extra method, we suggest that the method is a feasible option particularly to encourage the voices of adolescents and other marginalised populations.

Lastly, participants indicated that they preferred to engage in the dialogue in their first language, *isiZulu*. However, because not all the participants observers were bilingual, the facilitator switched between English and *isiZulu* resulting in much richness of the dialogue being lost (this became apparent from the content of discussions in the FGDs). In cases where bilingual community dialogues are unavoidable, it may be useful to have a bilingual note taker writing key points in English on a flip chart. This process was followed during the dialogue and was reportedly helpful to participants. It is recommended that the time allocated between sessions and overall time of the dialogue be closely monitored (and adjusted in subsequent dialogues) to avoid overly fatiguing participants.

## Conclusion

The community dialogue proved a feasible and enabling participatory approach for productive conversations between HCPs and the community on issues related to quality of care in the context of provision of FP/C services. We identify key elements that may be considered when constituting similar dialogues in the future. In particular, the role of the facilitator was identified as crucial to the success of the dialogue, as was the assistance of participant observers who acted as a support to the facilitator. Further, a clear framework to guide the discussion and nuanced timekeeping to keep the discussion dynamic and participants from tiring are critical. Lastly, ensuring adequate representation by carefully choosing timing and place of the dialogue are important as well as paying attention to equalising power differences between participants by creating a safe space for inclusive discussion.

Increasingly, community dialogues are being used in the South African health sector to encourage dialogue between community, civil society, health sector and other stakeholders. Our formative findings offer a systematic description and evaluation of a community dialogue process with key recommendations that may be considered when implementing similar community dialogues in the future.

## Additional file

Additional file 1: Focus Group Discussion Guide Community Dialogue. (DOCX 48 kb)

## Data Availability

The datasets used and/or analysed during the current study available from the corresponding author on reasonable request.
