# Supplementary material for: “As we have gathered with a common problem, so we seek a solution”: exploring the dynamics of a community dialogue process to encourage community participation in family planning/contraceptive programmes
Source: BMC Health Serv Res. 2019 Oct 17;19:710. doi: 10.1186/s12913-019-4490-6 (PMC6798361; doi:10.1186/s12913-019-4490-6)
Supplement: Supplementary file 1 — Focus Group Discussion Guide Community Dialogue. (DOCX 48 kb) [file 12913_2019_4490_MOESM1_ESM.docx]

| **FOCUS GROUP ID NUMBER:** | **LOCATION OF FGD:** | **DATE (DD/MMM/YY):** | **START TIME:** | **END TIME:** | **MODERATOR INITIALS:** |
| --- | --- | --- | --- | --- | --- |

**A HEALTH SECTOR AND COMMUNITY-BASED PARTICIPATORY APPROACH IN A HUMAN RIGHTS FRAMEWORK, TO INCREASE MET NEEDS FOR CONTRACEPTION: THE UPTAKE PROJECT**

**Focus Group Discussion Guide**

**Community Dialogue**

**UMKHAKHA WEZEMPILO NENDLELA YOKUBAMBA IQHAZA KOMPHAKATHI NGENDLELA YEZINHLELO ZAMALUNGELO ABANTU, UKWANDISA IZIDINGO ESEZITHOLAKELE ZOKUVIKELA UKUKHULELWA: I UPTAKE PROJECT**

**Uhla lwengxoxo yeqembu ehleliwe**

**Inkulumo Mpendulwano yomphakathi**

Introduction/ Isingeniso

*[Read to participant]/ [Fundela umbambiqhaza]*

We welcome all of you to this discussion. We are happy that you have spared some time to stay and have this discussion following the community dialogue. You have been invited to this discussion to provide your feedback on the community dialogue.

Siyanamukela nonke kulengxoxo. Siyajabula ukuthi nigcine (spared) isikhathi sokuhlala sibe nalengxoxo elandela inkulumo mpikiswano. Umenyiwe kulengxoxo ukuzonikeza izimpendulo (feedback) kwinkulumo mpendulwano.

*Purpose / Inhloso*

We are interested in all your views, ideas, comments, suggestions and recommendations. This research is to help us understand how to best engage community members and health care providers like you in order to develop an appropriate participatory intervention that will involve both community members and health care providers. The overall aim of the research project is to test whether an approach involving community and health care providers can increase met needs for family planning and contraception. All personal information will be treated with confidentiality.

Sithanda ukuzwa imibono, amazwi (comments), iziphakamiso kanye nezincomo. Lolucwaningo luzosisiza siqonde ukuthi singawabandakanya kanjani ngokuzimisela amalunga omphakathi kanye nabanikezeli bonakekelo lwezempilo njengawe ukuze sisungule ukungenelela kokubamba iqhaza okufanele okuzobandakanya kokubili amalunga omphakathi kanye nabanikezeli bonakekelo lwezempilo. Inhloso yonke yalolucwaningo ukuhlola ukuthi ingabe indlela enhlanganisa umphakathi kanye nabanikezeli bonakekelo lwezempilo ingazikhuphula izidingo esezifeziwe zokuhlela umndeni kanye nokuvukela ukukhulelwa. Lonke ulwazi lobunjalo lomuntu luyophathwa ngobumfihlo.

*Explain the ground rules for discussion/ Chaza isisuka mthetho salengxoxo*

This is an open discussion and there are no right or wrong answers, everyone’s point of view is important, so please feel free to discuss your opinions. We would like to have one person talk at a time and when one person is talking, there should be no interruption until the person has ended then the next person can share their views. There should be no side discussions. Anyone can contribute to the discussion at any time and everyone should feel free to disagree or agree with other views in a cordial manner. Please remember that what we discuss here today is confidential, do not discuss private information disclosed in this group with others outside of this group. Please turn cell phones on to silent so as not to disrupt the discussion. We will spend about one and a half to two hours for the discussion and some refreshment will be served at the end of the discussion.

We have just reviewed the consent form, which describes the study in detail and you have given us permission to speak with you in this group. As a reminder, you are not required to answer all of our questions, and you may skip any questions. As a reminder, we will use a digital recorder to record our conversation.

Do you have any questions before we begin the discussion?

Lena yingxoxo evulekile futhi ayikho impendulo okuyiyona noma okungeyona, yonke imibono yawowonke

umuntu ibalulekile, ngakhoke sicela ukhululeke ukuxoxa imbono yakho. Sizocela ukuba nomuntu oyedwa okhulumayo ngesikhathi, kumele kungabikhona kuphazamiseka aze aqede umuntu bese omunye umuntu olandelayo angabelana ngemibono yakhe. Noma ubani angabeka umbono engxoxweni noma ngasiphi isikhathi. Nonke ningakhululeka ukuphikisana noma ukuvumelana ngendlela enhle. Sicela ukhumbule ukuthi esikuxoxa la namhlanje kuyimfihlo, ungaluxoxi ulwazi oluyimfihlo olukhishwe kuleliqembu nabanye abangaphandle kuleliqembu. Sicela ucime umakhala ekhukhwini khona ungeke uphazamise ingxoxo. Sizothatha cishe ihora nohhafu kuya emahoreni amabili engxoxo futhi neziphuzo ziyotholakala ekupheleni kwengxoxo.

Siqeda kubuyekeza ipheshana lemvume, okuyilona oluchaza ucwaningo kabanzi futhi ususinikeze imvume yokhuluma nawe kuleliqembu. Njengesikhumbuzo, awubekelwe ukuthi uphendule yonke imibuzo yethu, futhi ungayeqa eminye imibuzo. Njengesikhumbuzo, sizosebenzisa isiqophamazwi ukuqopha ingxoxo yethu.

Ingabe unawo noma imuphi umbuzo ngaphambi kokuba siqale ingxoxo?

**Turn on digital recorder.**

I am (MODERATOR NAME) interviewing (FOCUS GROUP ID#) on [DATE] [START TIME]

|  | **Main question/Imibuzo** | **Probe/ Buzisisa** |
| --- | --- | --- |
| **Representation in the community dialogue/ Ukumelwa kwinkulumo mpendulwano yomphakathi** | | |
| 1.1 | Do you feel that all groups that make up the community in your district/area have been represented in this dialogue?  Ingabe uzwa/ucabanga ukuthi wonke amaqembu akha lomphakathi kwisifunda/indawo yakho amelwe kule nkulumompendulwano. | 1. Was there representation of minority groups or key populations? Describe who these were. / Ingabe bekukhona ukumelwa kwamaqembu abancane noma abantu okuyibonabona? Chaza bekungobani labo 2. Was there representation/representatives of adolescents? Describe who these were. / Ingabe bekukhona ukumelwa/abamele intsha? Chaza bekungobani labo. 3. Are there other groups that need to be engaged? Who are they and why do they need to be engaged? / Akhona amanye amaqembu adinga ukuhlanganyela? Obani labo futhi kungani bedinga ukuhlanganyela?   *Explore methods on how to engage groups that are not represented.*  *Hlola izindlela zokuthi angahlanganyela kanjani amaqembu angamelwanga.* |
| 1.2 | Was the health system represented?  Ingabe uhlelo lwezempilo belumelwe? | Are there other health system representatives who should be engaged? Who and Why? /Ingabe bakhona abanye abamele uhlelo lwezempilo okumele bahlanganyele? Obani futhi kungani?  *Explore how they can be effectively engaged.*  *Hlola bangahlanganyela kanjani ngokugcwele.* |
| **Establishing ground-rules/ Ukusungula isisuka-mthetho** | | |
| 2.1 | Did you understand the ground-rules of the community dialogue?  Ingabe uziqondile izisuka-mthetho zalenkulumompendulwano yomphakathi ? | 1. What do think was the purpose of setting grounds rules? Was this useful? How? / Ucabanga ukuthi yini inhloso yokuhlela isisuka-mthetho? Ngabe lokhu kusebenzile? Kanjani? 2. Were the rules explained in a way that was easy for you to understand? Which ones didn’t you understand, how could these be better explained? / Ingabe imithetho ichazwe ngendlela elula kuwe ukuthi uqonde? Yimiphi ongazange uyiqonde, ingachazwa kanjani kancono lena? |
| 2.2 | Did you agree with the ground-rules of the community dialogue?  Ingabe uvumelene nesisuka-mthetho senkulumompendulwano yomphakathi | *Probe for/Buzisisela*  *Explore points of agreement and disagreement. Hlola amaphuzu okuvumelana nokuphikisana*   1. Are there other ground-rules that should be included? What are they? / Ingabe ikhona eminye imisuka-mthetho okumele ibandakanywe? Imiphi yona? 2. Are there ground-rules that could have been removed? Which ones? / Ingabe zikhona izisusa-mthetho ebezingasuswa? Iziphi zona? |

| **Participation and dialogue in Community Dialogue/ Ukubamba iqhaza kanye nokuphendulana kwinkulumo mpendulwano yomphakathi** | | |
| --- | --- | --- |
| 3.1 | Did you feel at ease to communicate your views?  Ingabe uzizwe ukhululekile ukuxoxa imibono yakho? | *If they did not feel at ease, Explore why, and how they think this could be addressed e.g/ Uma bengazange bezizwe bekhululekile, hlola kungani, futhi bacabanga ukuthi kungalungiswa kanjani isb.*    *- How did you feel about expressing your views with elders/health workers/men/ community leaders present? / Uzizwe kanjani ngokuveza imibono yakho kukhona abadala/abasebenzi bezempilo/abesilisa/abaholi bomphakathi?* |
| 3.2 | Did the facilitators use language that you found appropriate and understandable?  Ingabe umphathingxoxo usebenzise ulimi oluthole lulungile futhi luqondakala? | *Explore reasons why yes or why not*  *Hlola izizathu kungani kunjalo noma kungani kungenjalo* |
| 3.3 | Did health care providers and community members discuss with each other?  Ingabe abanikezeli bonakekelo lwezempilo kanye namalunga omphakathi axoxile wodwa? | *Explore reasons why or why not. / Hlola izizathu kungani noma kungani kungenjalo?*  *If barriers were reported: Uma ukuvimbela/izithiyo bekubikiwe*  How can these be overcome? / Kunganqotshwa kanjani lokhu? |
| 3.3 | *[Ask in community members group and mixed group] [Buza kuqembu amalunga omphakathi kanye namaqembu exubile?]*  Did community members participate in the dialogue?  Ingabe amalunga omphakathi alibambile iqhaza kwinkulumo mpendulwano? | *Explore reasons why or why not. / Hlola izizathu kungani noma kungani kungenjalo.*  *If barriers were reported: uma ukuvimbela/izithiyo bekubikiwe:*  How can these be overcome? / Kunganqotshwa kanjani lokhu? |
| 3. 4 | *[Ask in health care providers group and mixed group] [Buza kubanikezeli bonakekelo lwezempilo Kanye neqembu elixubile]*  Did health care providers participate in the dialogue?  Ingabe abanikezeli bonakekelo lwezempilo balibambile iqhaza kwinkulumompendulwano? | *Explore reasons why or why not. / Hlola izizathu knagani noma kungani kunganjalo.*  *If barriers were reported: Uma ukuvimbela/izithiyo bekubikiwe:*  How can these be overcome?/ Kunganqotshwa kanjani lokhu? |

| **Discussing Quality of Care/ Ukuxoxa Ngezinga Lonakekelo** | | |
| --- | --- | --- |
| 4.1 | Was the topic of Quality of Care in relation to delivery of family planning/contraception discussed?  Ingabe isihloko sezinga lonakekelo besihlobene nokulethwa kokuhlela umndeni/ukuvikela ukukhulelwa okuxoxwe ngakho? | Do you understand what is meant by Quality of Care in FP?/ Ingabe uyaqonda ukuthi kushiwo ukuthini ngeZinga Lonakekelo ekuhleleni umndeni?  *Explore why or why not/ Hlola kungani noma kungani kungenjalo.*  If Quality of Care was not mentioned or not focused on, what was the main focus of this discussion?  Uma Izinga Lonakekelo alizange liphathwe noma kugxilwe kulo, bekuyini okuyiyonayona okugxilwe kuyo kulengxoxo? |
| 4.2 | Were Quality of Care definitions discussed?    Ingabe incazelo yezinga lonakekelo kuxoxiwe ngayo? | *Explore what definitions were discussed/*  *Hlola iziphi izincazelo okwaxoxwa ngazo* |
| 4.3 | Was there agreement between community and health care providers on the components of Quality of Care definition(s)?  Ingabe kwakukhona ukuvumelana phakathi komphakathi nabanikezeli bonakekelo lwezempilo ezingxenyeni zencazelo yezinga lonakekelo? | What are the main components that define Quality of Care? / Iziphi izingxenye okuyizonazona ezichaza izinga lonakekelo?    *Did health providers and community have different views about QoC in Family Planning/contraception? / Ingabe abanikezeli bonakekelo lwezempilo kanye namalunga omphakathi banemibono ehlukene ngezinga lonakekelo ekuhleleni umndeni nokuvikela ukukhulelwa?*  *If consensus is not reached, explore why? / Uma kwakungekho ukuvumelana, hlola kungani?* |
| 4.4 | *[Ask in community members group and mixed group][ Buza kwiqembu lamalunga omphakathi Kanye neqembu elixubile]*  As a community member, were you able to express your views on Quality of Care?  Njengelunga lomphakathi, ukwazile ukuveza imibono yakho kwiZinga Lonakekelo? | *Explore why or why not/ Hlola kungani noma kungani kungenjalo* |

| 4.5 | *[Ask in health care providers group and mixed group][ Buza kwiqembu labanikezeli bonakekelo lwezempilo Kanye neqembu elixubile]*  As health care providers, did you discuss Quality of Care in family planning/contraception?  Njengomnikezeli wonakekelo lwezempilo, uke waxoxa ngeZinga Lonakekelo ekuhleleni umndeni/ukuvikela ukukhulelwa? | *If yes, probe for: Uma ku-yebo buzisisela*   1. What did you think about the discussion? / Ucabangani ngengxoxo? 2. Was it a useful discussion in relation to your own work? / Ingabe bekuyingxoxo ewusizo ekuhlobaneni nomsebenzi wakho? 3. During the discussion, did you learn something new about QoC from the perspective of community members? What? How will this affect your work? / Ngesikhathi sengxoxo, khona okusha okufundile ngeZinga Lonakekelo kwimbono yamalunga omphakathi? Yini? Kuzowuthinta kanjani umsebenzi wakho lokhu?   *If not, probe for/ Uma kungenjalo, buzisisela*   1. What are the reasons why you did not discuss the topic? / Iziphi izizathu ezibangele ukuthi ungaxoxi ngesihloko? 2. Although you did not discuss QoC in this session, do you think it would still be useful for health care providers to discuss QoC in family planning/contraception with community members?/ Yize ungazange uxoxe ngeZinga Lonakekelo kulokhukuhlangana, ucabanga ukuthi kusabalulekile ukuthi abanikezeli bonakekelo lwezempilo baxoxe ngeZinga Lonakekelo ekuhleleni umndeni/ukuvikela ukukhulelwa namalunga omphakathi? |
| --- | --- | --- |
| **Views on Family Planning/Contraception/ Imibono ngokuhlela umndeni/ ukuvikela ukukhulelwa** | | |
| 5 | Do you agree that Family Planning/Contraception is an important issue to address?  Uyavumelana nokuthi ukuhlela umndeni/ukuvikela ukukhulelwa kuwudaba olubalulekile ukukhulunywa? | *If yes, explore the reasons why/ Uma yebo, hlola izizathu kungani*  *If no, explore the reasons why not/ Uma cha hlola izizathu kungani kungenjalo* |
| **Technical, schedule, cultural components of the approach/ Izigaba zobuchule, uhlelo, usiko lwendlela** | | |
| 6.1 | Were the terminologies used in the discussions explained?  Ingabe uhla lwamagama olusetshenziswe kwingxoxo lwachazwa? | *Explore which terminologies were difficult to understand./ Hlola iluphu uhla lwamagama olwalunzima ukuqondakala*  Were they addressed by the facilitators? How?/  Ingabe umphathingxoxo wakhuluma ngawo? Kanjani? |
| 6.2 | Were the materials (resources or tools) useful and did they initiate dialogue?  Ingabe izinsiza kusebenza (izinsiza kusebenza noma amathuluzi) asiza futhi ingabe ayiqalisa inkulumompendulwano? | *Explore reasons why and why not/ Hlola kungani futhi kungani kungenjalo* |

| 6.3 | Was the approach appropriate in your cultural context?  Ingabe indlela yayilungile ngokosiko lwakho? | *Explore reasons why and why not/ Hlola kungani noma kungani kungenjalo*  *Probe for: Buzisela*   1. *Traditional and religious beliefs/ Izinkolo zesintu nezinkolo zesonto* 2. *District or area of residence/ Isifunda noma indawo yokuhlala* 3. *Gender/ Ubulili* 4. *Others/ Okunye* |
| --- | --- | --- |
| 6.4 | Was the duration of the meeting appropriate?  Ingabe ubude bengxoxo bekulungile? | *Probe for/ Buzisisela*  Was it too long or too short? / Ingabe ibiyinde kakhulu noma imfishane kakhulu?  *Explore reasons why/ Hlola izizathu kungani?*   1. Was enough time given to all the participants to speak? / Ingabe isikhathi esanele sasinikeziwe bonke ababambiqhaza ukuba bakhulume? 2. Was enough time given to explaining the aims of the activity? / Ingabe isikhathi esanele sasinikeziwe ukuchaza inhloso yomsebenzi? 3. Was enough time given to explaining how the community dialogue worked? / Ingabe isikhathi esanele sasinikiwe ukuchaza ukuthi isebenza kanjani inkulumompendulwano? |
| 6.5 | Do you think there was the right number of people in the community dialogue meeting to have a discussion?  Ucabanga ukuthi bekunenani labantu elanale emhlanganweni wenkulumompendulwano kuze kube nenkulumo? | *Probe for: Buzisisela*   1. Were there too many people? What effect did it have to the meeting? / Ingabe abantu bebebaningi kakhulu? Kube namuphi umthelela kumhlangano? 2. Was there not enough people? What effect did it have on the meeting? / Ingabe abantu bebenganele? Kube namuphi umthelela kumhlangano?   *If not more than 70% of confirmed participants attended the meeting, explore possible reasons why. / Uma ababambiqhaza bengadlulanga ku 70% abafikile kwababeqinisekisile ukuza kumhlangano, hlola izizathu kungani?* |
| **Conclusion** | | |
| 7 | Do you have any other comments or questions?  Ingabe kukhona ukuphawula noma ikuphi onakho noma imibuzo? |  |

This is the end of our discussion. Thank you for your time.

Sekuyisiphetho sengxoxo yethu lesi. Siyabonga ngesikhathi sakho.
